# A New Method of Diagnosing Certain Corneal Affections

**Published:** 1891-08

**Authors:** 


A NEW METHOD OF DIAGNOSING CERTAIN CORNEAL AFFECTIONS.—F. T. Smith, M. D., uses Flourescein or Flourescin—they are closely related chemically, clinical effect about the same, almost insoluble in water, addition of bicarbonate sodium increases solubility, chemically incompatible with cocaine hydrochlorale, and closely related to resorcin. They are coal tar products. Uses the following formula:

R. Flourescein (or flourescin) gr. v.
Sodii bicarb, - - gr. iij ss.
Aqua purae, - - oz. ss.
M. et ft sol.

This solution produces no effect on healthy or inflamed eye unless there is abrasion of corneal epithelium. If abrasion of cornea be present a greenish discoloration appears. The application is painless—does not irritate the most sensitive eye.

This test is useful in locating foreign bodies in cornea. The discoloration lasts from one to six hours, and gradually disappears.—*Alabama Med. and Surg. Age.*

